# Integrated Transcriptomic and Metabolomic Analyses Reveal Physiological and Hepatic Metabolic Responses of Largemouth Bass (*Micropterus salmoides*) to Subacute Saline–Alkaline Stress

**DOI:** 10.3390/ijms262412091

**Published:** 2025-12-16

**Authors:** Bingbu Li, Mingyang Liu, Hailong Wan, Zengsheng Han, Heng Zhang, Guixing Wang, Wei Cao, Lize San, Yucong Yang, Yuqin Ren, Jilun Hou

**Affiliations:** 1 State Key Laboratory of Mariculture Biobreeding and Sustainable Goods, Beidaihe Central Experiment Station, Chinese Academy of Fishery Sciences, Qinhuangdao 066100, China; libb@bces.ac.cn (B.L.); liumy@bces.ac.cn (M.L.); 13333391041@163.com (H.W.); t61387173@126.com (H.Z.); wanggx@bces.ac.cn (G.W.); caow@bces.ac.cn (W.C.); sanlz@bces.ac.cn (L.S.); yangyc@bces.ac.cn (Y.Y.); 2 Hebei Key Laboratory of the Bohai Sea Fish Germplasm Resources Conservation and Utilization, Beidaihe Central Experiment Station, Chinese Academy of Fishery Sciences, Qinhuangdao 066100, China; 3 Bohai Sea Fishery Research Center, Chinese Academy of Fishery Sciences, Qinhuangdao 066100, China; 4 Ocean College, Hebei Agricultural University, Qinhuangdao 066100, China; 5 Hebei Key Laboratory of Nano-Biotechnology, Hebei Key Laboratory of Applied Chemistry, College of Environmental and Chemical Engineering, Yanshan University, Qinhuangdao 066004, China; hanzs@ysu.edu.cn

**Keywords:** *Micropterus salmoides*, saline–alkaline stress, transcriptomics, metabolomics, metabolic disruption

## Abstract

Freshwater scarcity makes saline–alkaline water essential for sustainable aquaculture. Current research has primarily focused on individual salinity or alkalinity effects on fish, with limited studies addressing their interactive effects. We found significant synergistic toxicity between salinity and alkalinity (*r* ≈ −0.950/−0.925) in largemouth bass (*Micropterus salmoides*), demonstrating higher salinity levels corresponding to lower 96-h median lethal concentration (96 h LC_50_) values for alkalinity, and vice versa. A subsequent 56-day subacute stress trial (salinity: 6‰; alkalinity: 20 mmol/L) assessed the impact on largemouth bass through growth efficiency, histopathology, biochemical assays, transcriptomics, and metabolomics, comparing a saline–alkaline group (SA) with a normal control group (NC). There were no significant differences in growth or survival between the SA and NC groups, but the SA group exhibited pathological changes in gill and liver tissues. Biochemically, the SA group exhibited elevated malondialdehyde, glutathione, and blood urea nitrogen levels, whereas glutathione peroxidase activity significantly decreased. Integrated transcriptomics and metabolomics analyses demonstrated that saline–alkaline stress disrupts lipid, amino acid, and steroid metabolism in largemouth bass, affecting steroid biosynthesis, fatty acid metabolism, glycerophospholipid metabolism, and cysteine and methionine metabolism pathways. Fish adapt by adjusting gene expression and metabolite levels to maintain metabolic balance. This study highlights adaptive mechanisms and applications for sustainable largemouth bass culture in saline–alkaline environments.

## 1. Introduction

Saline–alkaline water bodies represent a substantial proportion of global aquatic resources, with China alone encompassing approximately 46 million hectares [1]. Characterized by elevated pH, high salinity–alkalinity levels, and complex ionic compositions, most of these resources remain underutilized [2,3]. Climate change projections indicate a sustained worldwide trend toward environmental salinization and alkalization [4]. Meanwhile, as freshwater aquaculture space continues to diminish, the development and utilization of saline–alkaline water resources have become crucial for sustaining aquaculture production. Saline–alkaline aquaculture has major potential, with the identification and breeding of high-value salt-tolerant species emerging as a key research priority [1].

Generally, most fish struggle to maintain normal physiological functions in saline–alkaline environments, where salinity and alkalinity act as primary stressors [2,5]. To counteract saline–alkaline stress, fish expend substantial energy to maintain osmotic balance, diverting resources away from growth, and experience developmental delays [6]. Saline or alkaline stress significantly influences the physiological and biochemical parameters of fish. In African catfish (*Clarias gariepinus*), increasing salinity levels induce substantial alterations in hematological indices, immune responses, oxidative stress markers, and osmoregulatory mechanisms [7]. Similarly, when crucian carp (*Carassius auratus*) were cultured in water with 60 mmol/L alkalinity for 60 d, they developed gill oxidative stress and exhibited significant changes in ammonia metabolism-related parameters, including blood ammonia, blood urea nitrogen (BUN), and glutamine (Gln) levels [8]. Beyond their physiological effects, both saline and alkaline stress exert profound effects on transcriptional regulation and metabolic homeostasis in fish [9]. Recent transcriptomic studies in marine medaka (*Oryzias melastigma*) have revealed tissue-specific responses; the gills exhibit differential gene expression predominantly associated with signal transduction, immune regulation, and endocrine pathways, whereas the liver shows metabolic reprogramming focused on amino acid metabolism and protein synthesis, demonstrating distinct organ-specific adaptation strategies to saline stress [10]. Similarly, alkaline stress in largemouth bass (*Micropterus salmoides*) induces severe pathological alterations, including gill deformation and hepatocellular vacuolation, accompanied by significant transcriptional changes in the pathways involved in steroid biosynthesis, terpenoid backbone biosynthesis, extracellular matrix (ECM)-receptor interaction, and focal adhesion [1]. However, in practice, most saline–alkaline waters contain coexisting salinity and alkalinity stressors [11], prompting researchers to identify fish species suitable for such environments. For example, Zhou et al. [6] conducted chronic stress tests on Amur minnow (*Phoxinus lagowskii*) at 10‰ salinity and 5, 7, 9, and 11 mmol/L alkalinity, demonstrating its rapid acclimation and potential for large-scale saline–alkaline aquaculture. In studies on mandarin fish (*Siniperca chuatsi*), 96 h LC_50_ values for salinity and alkalinity were determined as 13.91‰ and 13.45 mmol/L, respectively. Subsequent chronic stress trials at 5‰ and alkalinity 8 mmol/L, combined with analyses of growth efficiency and gill histomorphology, confirmed its strong salinity–alkalinity tolerance and suitability for low-salinity alkaline aquaculture [12].

The largemouth bass, commonly known as California bass, is native to North America. Renowned for its delicate flesh and lack of intermuscular bones, it has gained popularity among Chinese consumers. In China, production is increasing annually, making it a major aquaculture species [13]. Largemouth bass demonstrate substantial environmental adaptability [14,15], positioning them as a highly promising species for aquaculture in saline–alkaline environments. Growth efficiency in saline–alkaline water varies among fish species. For example, mandarin fish (*Siniperca chuatsi*) cultured at low salinity (5‰) exhibited significantly enhanced growth compared with those in freshwater and low alkalinity (8 mmol/L) environments, a pattern similarly observed in large yellow croaker (*Larimichthys crocea*) [12,16]. Conversely, Nile tilapia (*Oreochromis niloticus*) [17] and Amur minnow (*Phoxinus lagowskii*) [6] showed no significant differences in growth efficiency relative to freshwater controls when subjected to low carbonate alkalinity (3 and 11 mmol/L, respectively), although tilapia experienced notably reduced survival rates. The largemouth bass can survive in water with salinity up to 10‰ [18,19]. Recent studies have confirmed its strong alkaline tolerance, demonstrating its potential as a promising candidate for saline–alkaline aquaculture [1]. Nevertheless, the potential synergistic toxicity of salinity and alkalinity in fish remains undocumented owing to the complexity of saline–alkaline aquatic environments.

This study systematically determined the 96-h median lethal concentration (96 h LC_50_) of salinity and alkalinity in largemouth bass. By regulating water salinity and alkalinity parameters, we revealed for the first time the interactive relationship between salinity-altered alkalinity 96 h LC_50_ and alkalinity-altered salinity 96 h LC_50_. Subsequently, an 8-week subacute saline–alkaline aquaculture experiment was conducted, integrating histopathological examination, physiological and biochemical indicators, transcriptomics, and metabolomics to analyze the physiological adaptation and molecular regulatory mechanisms of largemouth bass in response to subacute saline–alkaline stress. These findings provide a theoretical foundation for expanding aquaculture space in saline–alkaline waters and promoting fish farming in such environments.

## 2. Results

### 2.1. Median Lethal Concentration (96 h LC_50_) for Salinity and Alkalinity

**Figure 1:**
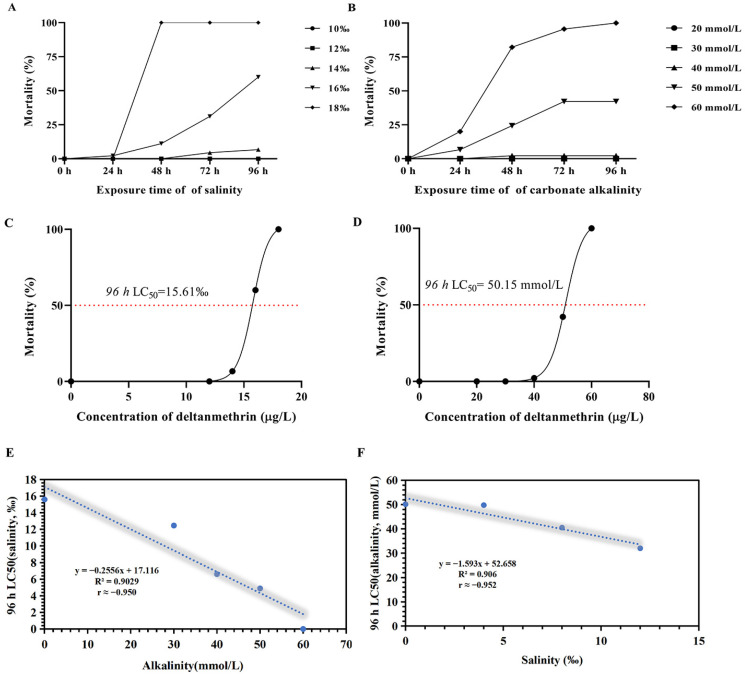
96 h acute exposure study in largemouth bass. (**A**) Effect of different salinity concentrations on the survival rate of largemouth bass; (**B**) effect of different alkalinity concentrations on the survival rate of largemouth bass; (**C**) 96 h LC_50_ of salinity stress for largemouth bass; (**D**) 96 h LC_50_ of alkalinity stress for largemouth bass; (**E**) linear regression equation between alkalinity and the salinity 96 h LC_50_; (**F**) linear regression equation between salinity and the alkalinity 96 h LC_50_.

The 96 h acute exposure tests showed that the lethal salinity concentration (96 h LC_100_) for largemouth bass was 18‰, with a safe level at 12‰ (Figure 1A), and the 96 h LC_50_ was 15.61‰ (Figure 1C). For alkalinity, the 96 h LC_100_ was 60 mmol/L, with a safe level of 30 mmol/L (Figure 1B), and the 96 h LC_50_ was 50.15 mmol/L (Figure 1D). Combined salinity–alkalinity stress tests revealed strong negative correlations between the 96 h LC_50_ values and the alternate stressor, indicating significant synergistic toxicity effects (Pearson correlation coefficient (r) ≈ −0.950/−0.925) (Figure 1E,F).

### 2.2. Effects of Salinity and Alkalinity Treatment on Growth Efficiency

The growth data are presented in Table 1. Analysis of the weight gain rate (WGR) showed higher values in the control group (NC), though the difference compared with the saline–alkaline stress group (SA) was not statistically significant (*p* = 0.109). A similar trend was observed for the specific growth rate (SGR), with the NC group displaying higher values than the SA group, but again without reaching statistical significance (*p* = 0.098). The survival rate (SR) was also higher in the NC group, though the difference was not significant relative to the SA group (*p* = 0.442). These results indicate that largemouth bass can survive long-term under specific saline–alkaline conditions without significant growth inhibition, thereby supporting the feasibility of saline–alkaline water aquaculture for this species.

### 2.3. Tissue Section Observation

**Figure 2:**
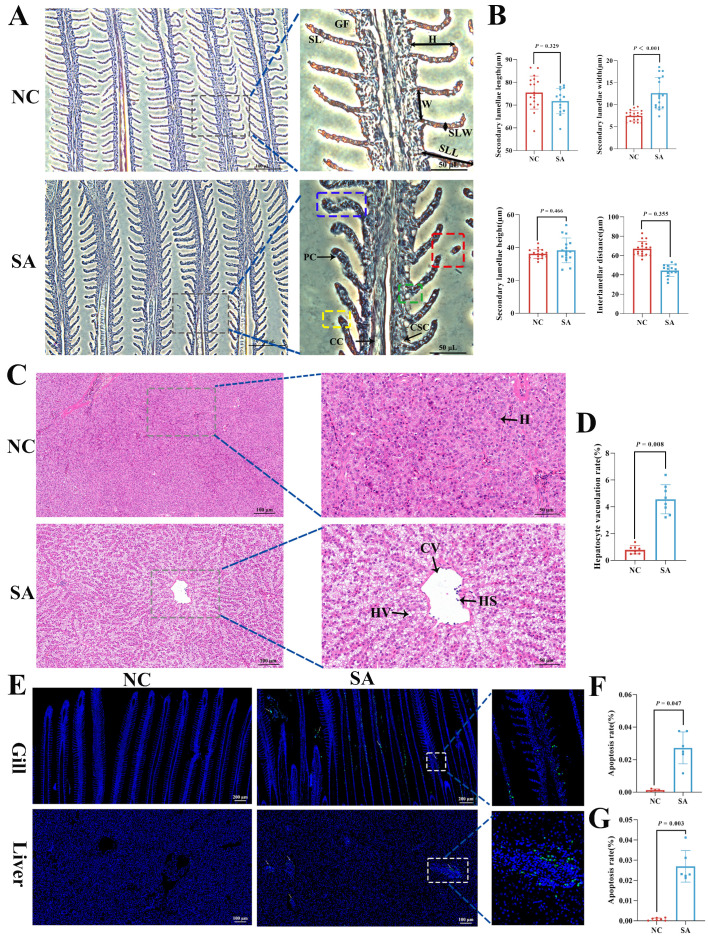
Histological sections of gill and liver tissues in largemouth bass. (**A**) HE-stained sections of gill tissue; (**B**) statistical analysis of secondary lamellae length, width, height, and interlamellar distance in gill tissue; (**C**) HE-stained sections of liver tissue; (**D**) statistical analysis of vacuolization rate in liver tissue; (**E**) TUNEL-stained sections of gill and liver tissues. Green fluorescence indicates a positive signal; (**F**) statistical analysis of apoptosis rate in gill cells; (**G**) statistical analysis of apoptosis rate in liver cells. GF: Gill filament; SL: secondary lamellae; SLL: secondary lamellae length; SLW: secondary lamellae width; SLH: secondary lamellae height; W: interlamellar distance; PC: pillar cells; H: hepatocytes; HV: hepatocyte vacuolization; HS: hepatic sinusoid; CV: central vein.

The results for the gill-stained sections are shown in Figure 2A. In the NC group, the secondary lamellae were arranged in an orderly manner, with tightly packed and neatly organized pillar cells. In contrast, the SA group exhibited several pathological alterations, including swelling at the tips of the secondary lamellae, disorganized arrangement of pillar cells (indicated by the blue box), detachment of the apical regions of the secondary lamellae (red box), erythrocyte shedding (yellow box), and hypertrophy and vacuolization of the chloride cells (green box). Moreover, statistical analysis demonstrated a significant increase in the width of the secondary lamellae (Figure 2B, *p* < 0.001).

Figure 2C shows the results for the liver-stained sections. The NC group exhibited intact liver structures with tightly packed hepatocytes and normal nuclei. In contrast, the SA group exhibited notable nuclear migration, severe cellular degeneration, and significant hepatocyte vacuolization (Figure 2D, *p* = 0.008).

TUNEL staining analysis of gill tissues demonstrated the absence of significant TUNEL-positive signals in the NC group. In contrast, pronounced TUNEL-positive signals were detected in the secondary lamellae of the SA group (Figure 2E), suggesting apoptosis in these areas. The SA group exhibited a significantly higher number of apoptotic cells than the NC group (Figure 2F, *p* = 0.047). Similarly, TUNEL staining of liver sections revealed a marked increase in apoptotic cells in the SA group relative to that in the NC group (Figure 2G, *p* = 0.003).

### 2.4. Analysis of Serum Physiological Parameters

**Figure 3:**
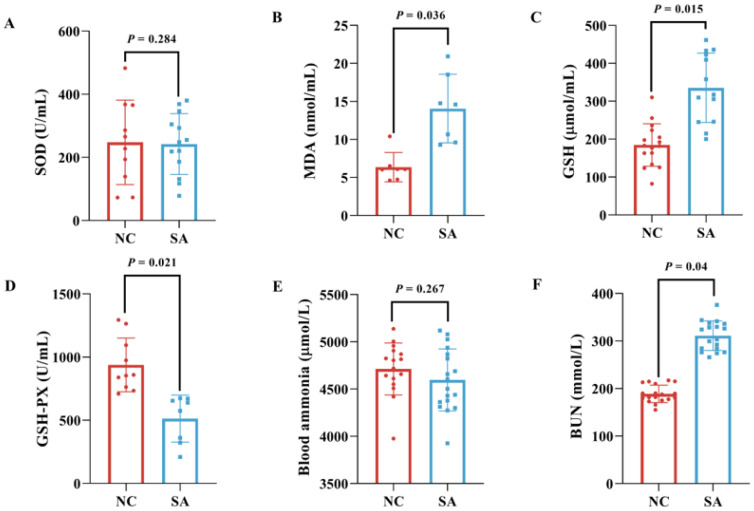
Impact of saline–alkaline stress on serum enzyme activity in largemouth bass. (**A**) SOD; (**B**) MDA; (**C**) GSH; (**D**) GSH-PX; (**E**) blood ammonia; (**F**) BUN, *n* ≥ 6.

Long-term subacute saline–alkaline stress significantly affected the antioxidant capacity of largemouth bass (Figure 3A–D). The SOD activity was not significantly different between the NC and SA groups (*p* = 0.284). MDA and GSH levels exhibited consistent trends, with both parameters being significantly elevated in the SA group compared with the NC group (MDA: *p* = 0.036; GSH: *p* = 0.015). In contrast, the GSH-PX activity demonstrated the opposite pattern, being significantly lower in the SA group than in the NC group (*p* = 0.021). These results indicate that the fish were experiencing oxidative stress. Although the increase in GSH represented a compensatory response, the impaired GSH-PX activity ultimately led to aggravated oxidative damage (as reflected by elevated MDA).

Furthermore, long-term subacute saline–alkaline stress disrupted the ammonia metabolism balance in the largemouth bass (Figure 3E,F). While blood ammonia levels showed no significant change in the SA group compared with the NC group (*p* = 0.267), BUN levels were significantly higher (*p* = 0.04).

### 2.5. Transcriptomic Analysis

The transcriptomic results (Table S1) showed that 677,509,738 raw reads were obtained from the liver tissue samples of the NC and SA groups, with 664,564,130 clean reads remaining after quality filtering.

**Figure 4:**
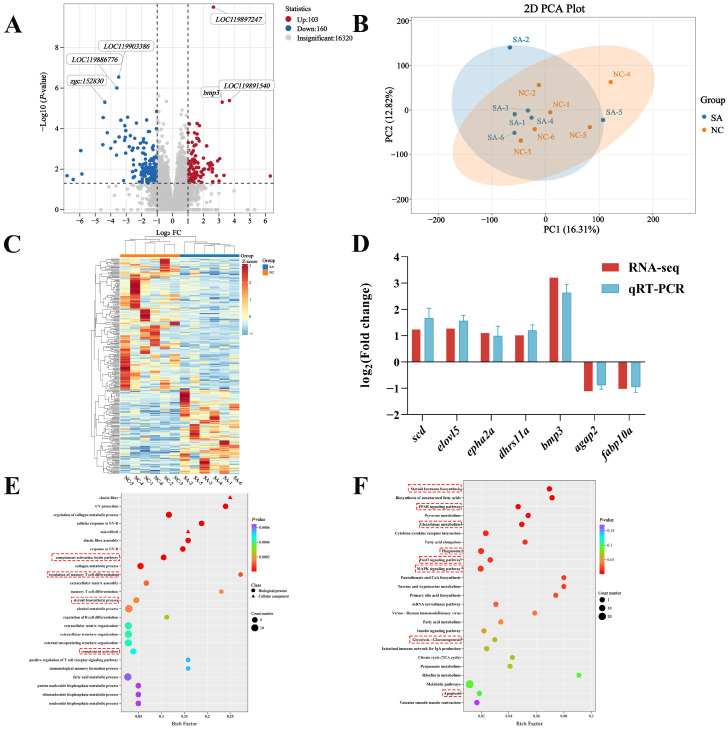
Transcriptome analysis of largemouth bass liver. (**A**) Volcano plot of DEGs; (**B**) PCA plot; (**C**) DEG clustering heatmap; (**D**) comparison of gene expression data between qRT-PCR and RNA-Seq; (**E**) GO enrichment analysis bubble plot. The red boxes indicated the key functional categories that were significantly enriched; (**F**) KEGG enrichment analysis bubble plot. The red boxes indicated the key signaling pathways that were significantly enriched.

In total, 264 DEGs were identified, including 103 upregulated and 161 downregulated genes (Figure 4A). PCA of the DEGs showed no clear separation between the SA and NC groups (Figure 4B). However, hierarchical clustering analysis revealed distinct expression patterns between the two groups, with clear separation and clustering into two distinct groups (Figure 4C). Moreover, qRT-PCR analysis of the DEGs demonstrated expression trends consistent with the transcriptomic data, confirming the reliability of the RNA-seq results (Figure 4D).

GO enrichment analysis demonstrated that 207 pathways were significantly enriched in the SA group compared to those in the NC group (Figure 4E, *p* < 0.05). Apoptosis was activated, whereas the pathways related to complement activation, steroid biosynthesis, and T/B cell regulation were significantly disturbed. The major enriched pathways included complement activation, lectin pathway (GO:0001867), collagen metabolic process (GO:0032963), regulation of memory T cell differentiation (GO:0043380), memory T cell differentiation (GO:0043379), steroid biosynthetic process (GO:0006694), regulation of B cell differentiation (GO:0045577), complement activation (GO:0006956), and positive regulation of T-cell receptor signaling pathway (GO:0050862).

KEGG enrichment analysis indicated that 16 pathways were significantly enriched in the SA versus NC comparison (Figure 4F; *p* < 0.05). Notable disruptions were observed in the glutathione, steroid, amino acid, and lipid metabolism pathways. The significantly enriched pathways included steroid hormone biosynthesis (ko00140), biosynthesis of unsaturated fatty acids (ko01040), PPAR signaling pathway (ko03320), pyruvate metabolism (ko00620), glutathione metabolism (ko00480), cytokine–cytokine receptor interaction (ko04060), fatty acid elongation (ko00062), phagosome (ko04145), FoxO signaling pathway (ko04068), MAPK signaling pathway (ko04010), taurine and hypotaurine metabolism (ko00430), pantothenate and CoA biosynthesis (ko00770), primary bile acid biosynthesis (ko00120), and fatty acid metabolism (ko01212).

### 2.6. Metabolomic Analysis

#### 2.6.1. Principal Component and Clustering in DMs

**Figure 5:**
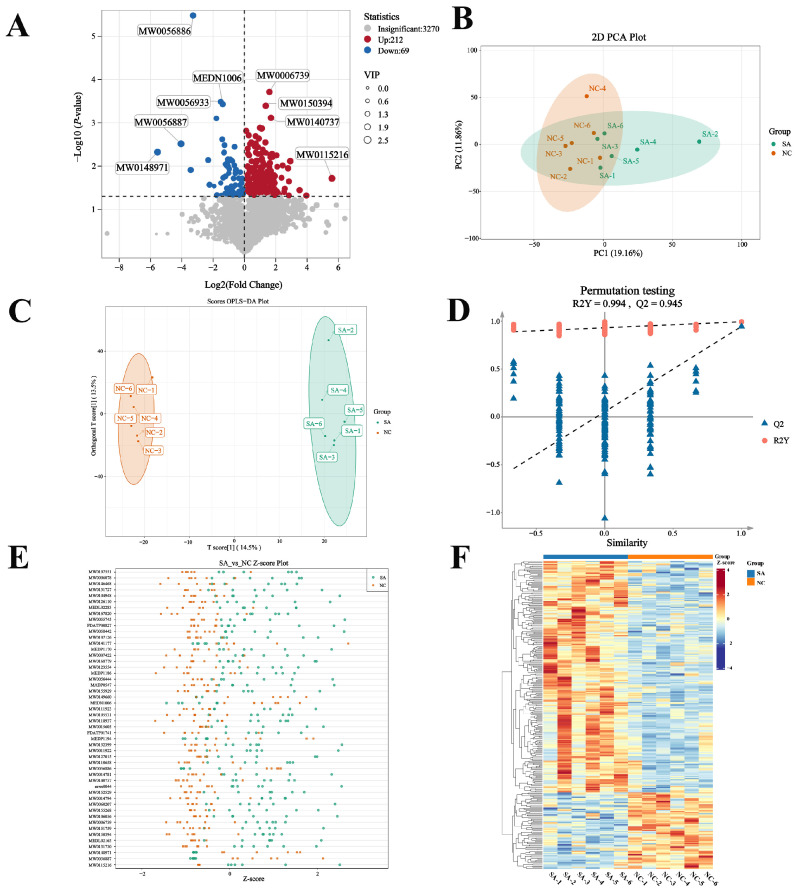
Hepatic metabolomic profiling of largemouth bass under saline–alkaline stress. (**A**) Volcano plot displaying differential metabolite distribution; (**B**) principal component analysis (PCA) score plot; (**C**) orthogonal partial least squares-discriminant analysis (OPLS-DA) score plot; (**D**) OPLS-DA model validation; (**E**) Z-score clustering of differential metabolites (DMs); (**F**) hierarchical clustering heatmap of DMs.

Volcano plot analysis for the comparison between SA and NA identified 281 DMs, of which 212 were upregulated and 69 were downregulated (Figure 5A). To provide a more intuitive representation of the differences between the sample groups, we conducted PCA and orthogonal partial least squares-discriminant analysis (OPLS-DA) of the SA and NC metabolites. PCA results indicated that metabolites from the SA and NC groups were not distinctly separated in the unsupervised analysis (Figure 5B). Although PCA is effective in extracting primary information, it is less sensitive to variables with weak correlations. Conversely, OPLS-DA, a supervised multivariate statistical method, is more adept at identifying variables with weak correlations. Unlike the PCA results, the OPLS-DA results demonstrated a clear separation between the SA and NC groups (Figure 5C), suggesting that saline–alkaline stress significantly affects liver metabolism. The OPLS-DA model was subsequently validated, yielding R^2^Y = 0.994 and Q^2^ = 0.945 (Figure 5D), indicating that the model was well-fitted, the data were robust, and further analyses could be performed reliably.

The Z-score plot and cluster heatmap of DMs (Figure 5E,F) demonstrated a distinct stratification between the saline–alkaline (SA) and normal control (NC) groups, indicating that saline–alkaline stress significantly influenced hepatic metabolic levels.

#### 2.6.2. KEGG Enrichment Analysis of DMs

**Figure 6:**
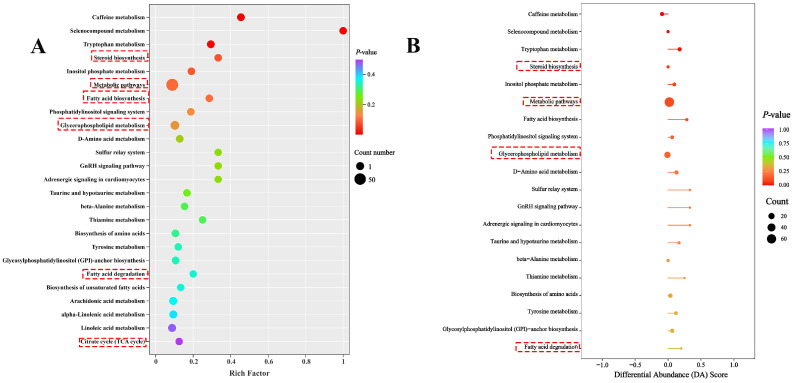
Metabolic pathway analysis of differential metabolites. The red boxes indicated the key signaling pathways that were significantly enriched. (**A**) KEGG enrichment analysis of DMs; (**B**) differential abundance score (DAS) pathway impact plot.

KEGG enrichment analysis revealed significant disruptions in amino acid and lipid metabolism, with a predominant trend of upregulation among the DMs (Figure 6A,B). The principal pathways enriched in this analysis included D-amino acid metabolism (ko00470); metabolic pathways (ko01100); caffeine metabolism (ko00232); selenocompound metabolism (ko00450); tryptophan metabolism (ko00380); inositol phosphate metabolism (ko00562); fatty acid biosynthesis (ko00061); phosphatidylinositol signaling system (ko04070); glycerophospholipid metabolism (ko00564); adrenergic signaling in cardiomyocytes (ko04261); the GnRH signaling pathway (ko04912); the sulfur relay system (ko04122); taurine and hypotaurine metabolism (ko00430); biosynthesis of unsaturated fatty acids (ko01040); tyrosine metabolism (ko00350); glycosylphosphatidylinositol (GPI)-anchor biosynthesis (ko00563); and fatty acid degradation (ko00071).

### 2.7. Integrated Analysis of Transcriptomics and Metabolomics

#### 2.7.1. KEGG Enrichment and Expression Trend Analysis

To better understand how saline–alkaline stress affects liver metabolism in largemouth bass, we analyzed transcriptomic and metabolomic data. Our KEGG-based joint enrichment analysis showed that both differentially expressed genes and metabolites in the SA vs. NC comparison were enriched in several pathways. Notably, disruptions were found in lipid metabolism, amino acid metabolism, and steroid biosynthesis, with key pathways including steroid biosynthesis, fatty acid biosynthesis, glycerophospholipid metabolism, and cysteine and methionine metabolism.

**Figure 7:**
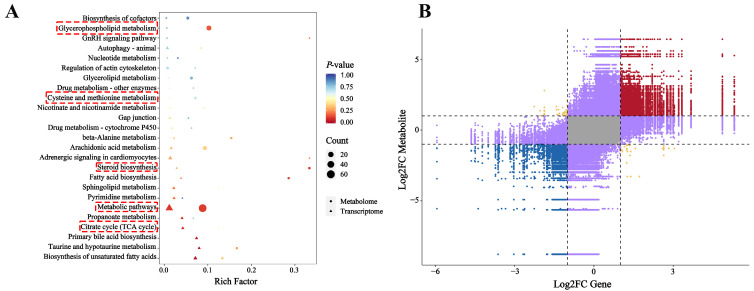
Integrated multi-omics correlation network. (**A**) KEGG pathway enrichment bubble diagram. The red boxes indicated the key signaling pathways that were significantly enriched; (**B**) nine-quadrant correlation analysis of genes and metabolites. Red and blue dots represented genes and metabolites with consistent expression patterns, both being either up- or down-regulated; yellow dots indicated opposite expression patterns; purple dots denoted no correlation between gene and metabolite expression; and the gray area included genes and metabolites that were not differentially expressed.

The nine-quadrant plot visualized fold-change patterns for substances with Pearson R > 0.80 and *p* < 0.05, in each comparison group, aiding in analyzing expression trends of DEGs and DMs in the SA vs. NC comparison. Most DEGs and DMs (in quadrants 3 and 7) showed consistent differential expression, suggesting positive gene regulation of DMs. Conversely, a few DEGs and DMs (in quadrants 1 and 9) displayed inconsistent patterns, indicating the possible negative gene regulation of these metabolites (Figure 7B).

#### 2.7.2. Correlation Analysis of DEGs and DMs

**Figure 8:**
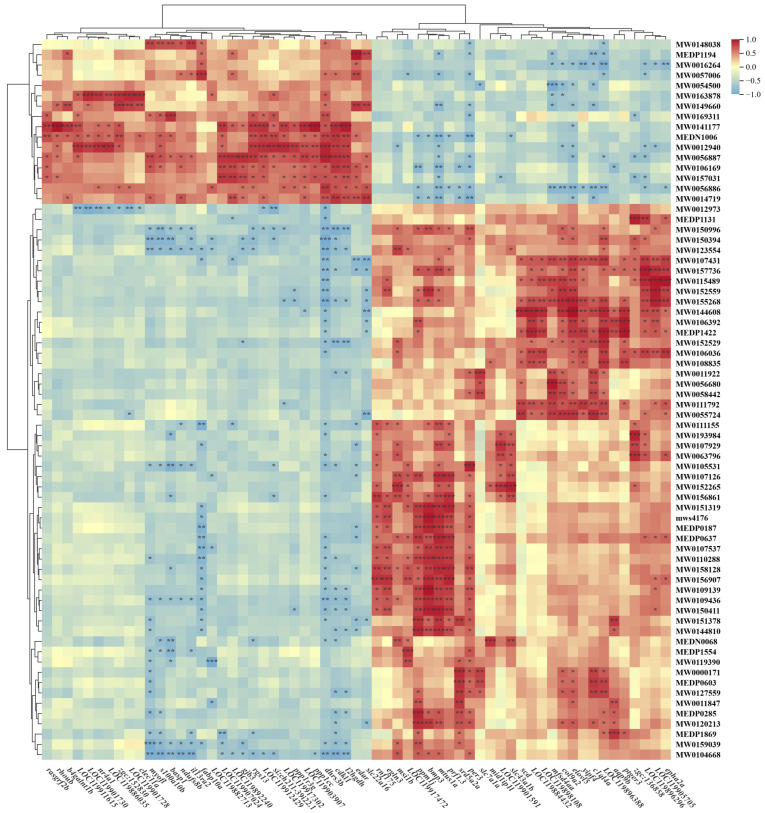
Correlation network analysis between differentially expressed genes and metabolites. Cross-omics correlation patterns between differentially expressed genes (DEGs) and differential metabolites (DMs). Asterisks denote statistical significance levels: * (*p* < 0.05), ** (*p* < 0.01), *** (*p* < 0.001).

To better understand the regulatory relationships between DEGs and metabolites (DMs) and comprehensively decipher the regulatory mechanisms underlying complex physiological processes, we filtered DEGs and DMs from the SA vs. NC comparison and generated a clustering heatmap to visualize their correlations, as shown in Figure 8.

Heatmap analysis revealed substantial correlations between genes and metabolites, indicating that saline–alkaline stress significantly influences amino acid metabolism in the fish liver. Multiple genes positively correlated with amino acids and their derivatives. For instance, in the cytokine–cytokine receptor interaction pathway, bone morphogenetic protein 3 (*bmp3*) exhibited strong positive correlations with MW0156907 (Ser-Ser-Glu, r = 0.85), MW0109139 (phenylalanyltyrosine, r = 0.94), MW0109436 (Pro-Thr, r = 0.85), and MW0110288 (tyrosylleucine, r = 0.84). *LOC119884432* was strongly correlated with MW0144608 (Ala-Ile-Asn, r = 0.89) in the glutathione metabolism pathway, whereas *agap2* showed a strong positive correlation with MW0148038 (Cys-Lys-Met, r = 0.85) in the FoxO signaling pathway.

Saline–alkaline stress significantly perturbs lipid metabolism in fish, as evidenced by the strong positive correlations observed between lipid-metabolism-related genes and metabolites across multiple pathways. Specifically, stearoyl-CoAdesaturase (*scd*) demonstrated strong positive correlations with MW0055724 (PA, r = 0.82) and MW0144608 (r = 0.87) across several metabolic pathways, including the biosynthesis of unsaturated fatty acids, metabolic pathways, fatty acid metabolism, and the PPAR signaling pathway. Similarly, fatty-acid-binding protein 10a (*fabp10a*) exhibited a significant positive correlation with MW0057006 (PC, r = 0.84), specifically within the PPAR signaling pathway. Furthermore, elovl fatty acid elongase 5 (*elovl5*) expression significantly correlated with multiple metabolites: MW0155268 (Phe-Lys-Gln, r = 0.88), MW0107431 (Ile-Asp-Lys, r = 0.81), MW0055724 (r = 0.83), MW0144608 (r = 0.95), MEDP1422 (Carnitine C8-OH, r = 0.81), and MW0106392 (D-glutamic acid, r = 0.81). These correlations were identified within several key lipid metabolic pathways, including fatty acid elongation, unsaturated fatty acid biosynthesis, metabolic pathways, and fatty acid metabolism.

## 3. Discussion

Saline–alkaline waters are globally prevalent; however, most freshwater fish species are unable to thrive under such conditions [20]. The development of suitable species for saline–alkaline aquaculture represents a critical initiative for enhancing the utilization efficiency of saline–alkaline water resources and expanding the aquaculture space. Current research predominantly focuses on the individual effects of either salinity or alkalinity on aquatic animals [1,19], while natural saline–alkaline waters typically feature complex compositions with coexisting salinity and alkalinity stressors [11]. Therefore, this study focused on the interaction between salinity and alkalinity to develop universally adaptable species for saline–alkaline aquaculture to facilitate the expansion of aquaculture operations. In this study, the largemouth bass exhibited significant salt tolerance, with a 96 h LC_50_ for salinity measured at 15.61‰. Additionally, previous research has demonstrated that largemouth bass possess considerable alkaline tolerance, with a reported 96 h LC_50_ of 41.41 mmol/L [1]. In the present study, the 96 h LC_50_ for alkalinity was slightly higher, at 50.15 mmol/L, potentially due to differences in fish size (23.60 ± 4.07 g vs. 9.72 ± 0.79 g). Our study revealed a significant correlation between salinity and alkalinity, demonstrating a strong synergistic toxic effect. Specifically, higher salinity levels correspond to lower 96 h LC_50_ values for alkalinity, and vice versa.

Environmental stressors can damage fish gills and liver [21,22,23]. Stress induced by both salinity and alkalinity results in differential degrees of hepatic damage in largemouth bass, which is characterized by deformations, displacement of hepatocyte nuclei, and vacuolization [1,24]. This study revealed that subacute saline–alkaline stress (salinity: 6‰; alkalinity: 20 mmol/L) induced both gill and liver damage, along with the occurrence of cellular apoptosis. Apoptosis, a well-established, genetically regulated process of programmed and orderly cell death, facilitates adaptation to adverse environmental conditions and plays a crucial role in phylogeny and tissue remodeling [25,26]. Transcriptomic analysis of the liver revealed the enrichment of multiple genes in apoptosis-related pathways. Notably, the transcript levels of the perforin Ala91Val (*prf1.3*) gene were significantly elevated, demonstrating a strong positive correlation (R > 0.80) with several metabolites, including MW0107537 (indole), MW0151319 (Ile-Ala-Tyr), and MW0158128 (Trp-Gln-Gln). The *prf1.3* gene represents a mutant variant (c.272C > T) of perforin (*prf1*), which is implicated in the activation of apoptotic pathways and promotion of programmed cell death [27]. The Ala91Val mutation has been documented to result in the dysregulation of immune activation [28]. Therefore, *prf1.3* may play a critical role in cellular apoptosis.

Saline–alkaline stress notably affects serum immune function and antioxidant capacity [8]. Elevated levels of MDA, a lipid peroxidation byproduct [29], indicate oxidative stress in fish. GSH-Px boosts the antioxidant capacity by converting reduced GSH to oxidized glutathione (GSSG) [30,31]. This study found that long-term saline–alkaline stress significantly reduced GSH-Px activity and increased MDA levels in serum, consistent with findings observed in crucian carp under chronic alkaline stress [8]. Transcriptomic analysis has shown enrichment of oxidative stress-related pathways, particularly the MAPK signaling pathway, which is activated by reactive oxygen species (ROS) and modulates inflammatory responses to alleviate oxidative stress [32,33]. In this study, serum GSH activity was significantly increased, and transcriptomic analysis showed notable enrichment of DEGs in the glutathione metabolism pathway, which is crucial for cellular defense against oxidative stress and immune regulation [34]. Among these, *LOC119884432*, which encodes a phospholipid hydroperoxide glutathione peroxidase-like protein, was highly expressed and was positively correlated with several metabolites, including Ala-Ile-Asn, MW0138445 (irigenin), and MEDP1422 (carnitine C8-OH). We hypothesized that the observed increase in GSH activity in fish was associated with the upregulated expression of *LOC119884432*, which may contribute to the maintenance of cellular function under stress conditions [35].

Saline–alkaline stress not only causes oxidative stress and inflammation but also disrupts metabolic processes in aquatic organisms, particularly affecting steroid and lipid metabolism [9]. In the largemouth bass, it disturbs steroid biosynthesis and lipid metabolism, potentially causing alkalosis [1]. This study found similar disruptions in steroid and lipid metabolism under long-term saline–alkaline conditions, along with imbalanced urea cycle regulation. Elevated BUN levels indicated impaired urea metabolism, with significant enrichment of urea cycle metabolites such as L-ornithine and urea. Additionally, pathways such as steroid hormone biosynthesis, glycerophospholipid metabolism, arachidonic acid metabolism, fatty acid biosynthesis, and other metabolic pathways were enriched among DEGs and DMs. Steroid hormone biosynthesis is a key hepatic pathway that converts cholesterol to bioactive hormones [36]. Glycerophospholipids are essential for cell membrane structure, and their metabolic disruption can harm membrane integrity [37,38]. This study found that *scd*, *fabp10a*, and *elovl5* were positively correlated with various phospholipids (e.g., PA and PC) and amino acids in lipid metabolism pathways. Notably, *scd* regulates monounsaturated fatty acid (MUFA) synthesis for membrane function [39], whereas *fabp10a* and *elovl5* influence lipid metabolism [40,41]. We suggest that long-term saline–alkaline aquaculture disrupts lipid metabolism in largemouth bass, prompting compensation through the regulation of *scd*, *fabp10a*, and *elovl5* to restore metabolic balance.

## 4. Materials and Methods

### 4.1. Experimental Design

#### 4.1.1. Experimental Condition

The experimental subjects were largemouth bass fry bred at the Beidaihe Central Experimental Station of the Chinese Academy of Fishery Sciences. A cohort of 1000 healthy and active fry, with an average weight of 23.60 ± 4.07 g and an average length of 9.20 ± 0.78 cm, was selected for temporary culture in a 50 m^3^ concrete tank. For the acute toxicity challenge experiment, 300 L culture tanks were utilized. Prior to the commencement of the experiment, the fry were given a 15-day acclimation period in culture tanks. The culture water used was filtered tap water, aerated for 48 h, and maintained at a temperature of 23 ± 1 °C. Each aquarium was equipped with two airstones to ensure that the DO levels remained above 4 mg/L. During the acclimation period, the fish were fed once daily with commercial feed (Haida, Dongguan, China) at a rate of 1% of their total body weight. Water exchange was conducted every 24 h, with an 80% replacement volume.

#### 4.1.2. Acute Stress Experiment

Acute salinity stress experiment: salinity levels were established at 0, 10, 12, 14, 16, and 18‰, with 0‰serving as the control group (NC). Saline-water solutions were prepared by mixing seawater with freshwater. Three replicate experimental tanks were established for each treatment group, each with a volume of 240 L. Fifteen fish were placed in each tank. Water temperature, dissolved oxygen, ammonia, nitrogen, nitrite, and salinity were monitored daily. Feeding was suspended throughout the experimental period, the water was renewed every 24 h, and fish mortality was recorded.

In the acute alkalinity stress experiment, the alkalinity levels were set at 0, 20, 30, 40, 50, and 60 mmol/L using Na_2_CO_3_ and NaHCO_3_ in a 1:16.1 mass ratio. Daily alkalinity measurements were performed, maintaining the same conditions as in the acute toxicity experiment, with equivalent salinity. The 96 h LC50, with a 95% confidence interval, was determined using the probit method.

In the saline–alkaline acute toxicity test, the 96 h LC_50_ for fish fry was 15.61‰ salinity with a safe level of 12‰, and 50.15 mmol/L alkalinity with a safe level of 30 mmol/L. Combined tests assessed interactions, measuring alkalinity LC_50_ at salinities of 0, 4, 8, and 12‰, and salinity LC_50_ at alkalinities of 0, 30, 40, 50, and 60 mmol/L, using the same methods as individual tests.

#### 4.1.3. Subacute Saline–Alkaline Stress Experiment

The experiment involved two groups: a freshwater control group and a saline–alkaline stress group (salinity: 6‰, alkalinity: 20 mmol/L). Using the method described in Section 4.1.2, salinity and alkalinity were adjusted, and 90 healthy juvenile largemouth bass were placed in six 300 L tanks. Over 56 days, the fish were fed twice daily, with daily monitoring of water temperature, salinity, and alkalinity, while maintaining water quality and management, as described in Section 4.1.2.

### 4.2. Sample Collection

Following the subacute-exposure experiment, samples were collected after a 24 h fasting period. Each group of fish was anesthetized using 100 mg/L MS-222 (Sigma Aldrich, GYT0202813, St. Louis, MO, USA), and growth indicators such as weight gain rate (WGR, %), specific growth rate (SGR, %), and survival rate (SR, %) were measured and evaluated [42].

Nine fish from each group were randomly selected for arterial blood collection. After 12 h, serum was collected for enzyme activity analysis. Gill and liver tissues were fixed in 4% paraformaldehyde for stained section preparation. Liver tissue samples were collected from six individuals per group and promptly cryopreserved in liquid nitrogen for subsequent transcriptomic and metabolomic analyses.

### 4.3. Histological Processing

#### 4.3.1. HE-Staining Observation

Paraffin-embedded sections were prepared according to the method described by Zheng et al. [43]. The fixed tissues were dehydrated in an ethanol gradient, washed with a mixture of equal amounts of xylene and anhydrous ethanol, embedded in wax using a Leica EG1150H (Leica, Wetzlar, Germany), and sliced into thin sections using a Leica RM2255 (Leica, Wetzlar, Germany). The sections were dewaxed using ethanol and xylene and sealed with neutral gum after hematoxylin and eosin staining. Finally, the sections were examined and photographed using a Leica DM3000 microscope (Leica, Wetzlar, Germany). Morphometric parameters, including secondary lamellae length, width, height, and interlamellar distance in gill tissue as well as the hepatic vacuolation rate, were measured and calculated using Image-Pro Plus 6.0 software [1].

#### 4.3.2. TUNEL Detection

Apoptosis was detected using the TUNEL method, which stains the liver and gill tissues with green fluorescence to detect apoptotic cells [44]. Gill and liver paraffin sections were placed in an eco-friendly dewaxing solution (Sevicebio, G1128, Wuhan, China) for 10 min, the solution was replaced, and the process was repeated three times. Dewaxed tissue sections were immersed in anhydrous ethanol for 5 min, and the solution was replaced three times. Following dehydration, the sections were outlined, covered with proteinase K solution, and incubated at 37 °C for 20 min. Next, the sections were washed three times with PBS (5 min per wash), followed by a brief drying step, and incubated with buffer at room temperature for 10 min. The TUNEL assay reaction solution (Servicebio, G1504, Wuhan, China) was prepared by mixing the TdT enzyme, dUTP, and buffer in a 2:5:50 ratio and then applying it to the tissue. The sections were then incubated in a humidified chamber at 37 °C for 1 h. The sections were then washed with PBS and gently shaken to remove excess liquid. Subsequently, 2,6-diaminopyridine (DAPI) staining solution (Servicebio, G1012, Wuhan, China) was applied dropwise within the designated area, and the samples were incubated in the dark for 10 min. After incubation, the sections were sealed with an anti-fluorescence quenching agent. The stained sections were observed using a Nikon Eclipse C1 fluorescence microscope (Nikon, Tokyo, Japan) and scanned using a Pannoramic MIDI scanner (Nikon, Tokyo, Japan). Detection and quantification of the apoptosis rate were performed with Image-Pro Plus 6.0 software [1].

### 4.4. Serum Enzyme Activity

The concentrations of blood ammonia (Jiancheng, A086-2-1, Nanjing, China) and the enzymatic activities of superoxide dismutase (SOD; Jiancheng, A001-3-2, China), malondialdehyde (MDA; Jiancheng, A003-1-2, China), glutathione (GSH; Jiancheng, A006-2-1, China), glutathione peroxidase (GSH-PX; Jiancheng, A005-1-2, China), and blood urea nitrogen (BUN; Jiancheng, C013-1-1, China) in serum were quantified utilizing commercial assay kits procured from the Nanjing Jiancheng Bioengineering Institute, Nanjing, China.

### 4.5. Transcriptomics

#### 4.5.1. Transcriptome Sequencing of Largemouth Bass Liver Samples

Total RNA of liver tissues was extracted using an Animal Tissue Total RNA Kit (Tiangen, DP431, Beijing, China), and after RNA integrity was confirmed using a 1% agarose gel, RNA purity (OD260/280) and concentration were measured using a NanoDrop 2000 (Thermo, Waltham, MA, USA) and then stored in the refrigerator at −80 °C until transcriptome sequencing (RNA-seq) and real-time quantitative fluorescence PCR (qRT-PCR) analysis. Twelve samples were prepared (NC-1, NC-2, NC-3, NC-4, NC-5, NC-6, SA-1, SA-2, SA-3, SA-4, SA-5, and SA-6). Polyadenylated mRNAs were enriched using oligo(dT) magnetic beads, randomly fragmented, and libraries were created [45]. Once the library check was completed, the libraries were combined and sequenced using Illumina XPlus (Illumina, San Diego, CA, USA).

#### 4.5.2. Data Analysis

Fastp removed low-quality reads, and Hisat2 indexed and aligned the cleaned reads to the largemouth bass genome [46]. StringTie constructed transcripts from these alignments [47], and gene expression was calculated using fragments per kilobase of exon model per million mapped fragments (FPKM) values [48]. DESeq2 identified differentially expressed genes with a false discovery rate of <0.05, and |log2(fold change)| > 1 [49]. GO and KEGG pathway enrichment analysis of these genes was conducted using clusterProfiler [50], with the results displayed as scatter plots.

#### 4.5.3. Quantitative Real-Time PCR

The expression of 12 differentially expressed genes (DEGs; Table 2) was analyzed using qRT-PCR, with the *β*-actin gene amplification as the internal control [48]. High-quality total RNA was isolated and used to synthesize complementary DNA. The PCR reaction system totaled 10 μL, including 5 μL TB Green Premix Ex Taq II (Takara, RR820A, Kyoto, Japan), 0.5 μL each of forward and reverse primers, and 0.5 μL cDNA. The procedure included pre-denaturation at 94 °C for 2 min, 40 cycles of denaturation for 30 s, annealing at 60 °C for 20 s, and a final extension at 65 °C for 5 s and 95 °C for 5 s. Product specificity was verified via the melting curve, and mRNA expression levels were determined using the 2^−ΔΔCt^ method [51].

### 4.6. Metabolomic

#### 4.6.1. Sample Processing

Liver samples were thawed from a −80 °C freezer, minced, and homogenized. The samples were then placed in centrifuge tubes with sterilized steel beads and homogenized in a cryogenic grinder for 90 s. After centrifuging at 3000 r/min for 30 s at 4 °C, 400 μL of 70% methanol was added. The mixture was shaken at 1500 r/min for 5 min and incubated on ice for 15 min. Samples were then centrifuged at 12,000 r/min for 10 min, and 300 μL of the supernatant was transferred to a new tube. This tube was centrifuged again for 10 min, and 200 μL of the supernatant was collected for liquid chromatography–mass spectrometry (LC-MS) analysis.

#### 4.6.2. LC-MS Conditions

All samples were analyzed by LC-MS in both positive and negative ion modes. In positive ion mode, the samples were eluted using a T3 chromatographic column (Waters, 186009469, Milford, MA, USA). The mobile phase system was composed of two components: mobile phase A, consisting of 0.1% formic acid in water, and mobile phase B, consisting of 0.1% formic acid in acetonitrile. The gradient elution program was structured as follows: from 0 to 2 min, the proportion of phase A was linearly decreased from 95% to 80%, whereas the proportion of Phase B increased from 5% to 20%. Between 2 and 5 min, phase A was further decreased from 80% to 40%, with a corresponding increase in phase B from 20% to 60%. Subsequently, within 1 min, phase A was rapidly reduced to 1% and phase B increased to 99%, maintaining this ratio for 1.5 min. Finally, phase A was swiftly restored to 95% and phase B was reduced to 5% within 0.1 min, followed by a 2.4 min equilibration period. The analysis was conducted under the following parameters: a column temperature of 40 °C, a flow rate of 0.4 mL/min, and an injection volume of 4 μL. The chromatographic conditions and gradient elution program for negative ion mode were identical to those for positive ion mode, with the sole exception of the direction of ionization polarity. Mass spectrometric detection employed an alternating mode of full scan (*m*/*z* 75–1000, resolution 35,000) and data-dependent multistage fragmentation, utilizing collision energies of 30, 40, and 50 V, in conjunction with a dynamic exclusion strategy set at 3 s. Detailed parameters are provided in Supplementary Table S1.

#### 4.6.3. Data Processing and Analysis

The initial mass spectrometry data were transformed into the mzML format using ProteoWizard. Subsequent steps involved peak extraction, alignment, and retention time correction, all of which were conducted using XCMS plus software (https://xcmsonline.scripps.edu/, accessed on 3 February 2025). The resulting filtered peaks were annotated by searching against an in-house laboratory database, public databases, and predictive libraries, including the Human Metabolome Database (http://www.hmdb.ca/, accessed on 3 February 2025) and LIPID MAPS (http://www.lipidmaps.org/, accessed on 3 February 2025). Finally, a comprehensive data matrix was constructed encompassing mass-to-charge ratio (*m*/*z*), retention time (RT), peak intensity, sample name, and metabolite identification.

The collected data underwent unit variance scaling before being analyzed through principal component analysis (PCA) using the ‘prcomp’ function from the R statistical package (www.r-project.org, accessed on 3 February 2025). Hierarchical clustering and Pearson correlation coefficient analyses of metabolites were conducted using the ComplexHeatmap package in R [52], and the results are displayed as heatmaps. The orthogonal projections to latent structure-discriminant analysis (OPLS-DA) model was used to identify differentially expressed metabolites (DMs). Metabolites were classified as DMs if they satisfied the criteria of *p*-values less than 0.05 (as determined by Student’s *t*-test) and variable importance in projection (VIP) values greater than 1.0, from the OPLS-DA model. The reliability of the model was confirmed through 200 permutation tests, with R^2^Y values less than 0.3 and Q^2^ values less than 0.05, indicating satisfactory validation. Finally, identified metabolites were annotated using KEGG Compound database (http://www.kegg.jp/kegg/compound/, accessed on 3 February 2025), and the annotated metabolites were then mapped to the KEGG Pathway database (http://www.kegg.jp/kegg/pathway.html, accessed on 3 February 2025).

### 4.7. Integrated Transcriptome and Metabolome Analysis

DMs (*p* < 0.05 and VIP > 1) and DEGs (*p*-adjust < 0.05 and |log2FC| ≥ 1) were used for integrative analysis of control and SA groups. The Pearson method was used to analyze the correlation coefficients of the data between metabolomics and transcriptomics using the R package (ComplexHeatmap) [52]. A heatmap was used to show the connections between the DMs and DEGs.

### 4.8. Statistics and Analyses

The experimental data are presented as the mean ± standard error. All data were processed utilizing the R software package (beadarray) [53] and analyzed for enzyme activity and qRT-PCR data using an independent samples *t*-test to identify significant differences between groups.

## 5. Conclusions

This study confirms the synergistic toxicity of salinity and alkalinity on fish, while demonstrating that largemouth bass can be cultured in low-saline–alkaline water (salinity: 6‰; alkalinity: 20 mmol/L) with no significant differences in growth or survival rates compared with freshwater aquaculture. However, long-term saline–alkaline culture was found to cause gill and liver damage, leading to oxidative stress and hepatic lipid metabolism disorders. Therefore, achieving sustainable saline–alkaline aquaculture of largemouth bass requires further breeding of salt–alkaline-tolerant strains to expand the aquaculture space. This study provides theoretical support for the saline–alkaline aquaculture of largemouth bass and offers new insights for future breeding efforts in saline–alkaline environments.

## Figures and Tables

**Table 1:** Growth efficiency of largemouth bass.

Project	Groups		*p*-Value
	NC	SA	
WGR (%)	131.45 ± 8.277	105.29 ± 19.271	0.109
SGR (%/d)	1.50 ± 0.065	1.27 ± 0.161	0.098
SR (%)	88.89 ± 1.283	81.19 ± 1.960	0.442

**Table 2:** Gene qRT-PC primer information.

Primers	Forward (5′-3′)	Reverse (5′-3′)
*scd*	TGACCTGTACGCCGACAAAG	ATGAAGTACGCCACCCACAG
*fabp10a*	CAGGGCCATGGGAATGAACA	CTTTCCAGGGGTCTTGGAGG
*elovl5*	CCCTGTGGCCATTCGTACTT	TCTTCCACCAAAGGTACGGC
*bmp3*	AAGCCCTCTAACCATGCCAC	ACCACGTTCTTGTCCTCGTC
*agap2*	TGCTACTGATCCGGGAGGAA	TAAAGCTCCTGGAAGCTGGC
*epha2a*	TCGCCGTAGCGATTAAGACC	TGGCGTGCTTGAATTTGGTG
*dhrs11a*	TGCTCAGCGGAAAGACAGAG	AGTGGCGCAGTAGAAATGCT

## Data Availability

The data are contained within the article. RNA-seq data are submitted in China National Center for Bioinformation (BioProject: CRA033393).

## Supplementary Materials

The following supporting information can be downloaded at: https://www.mdpi.com/article/10.3390/ijms262412091/s1.

## References

[1] Hua J.X., Tao Y.F., Lu S.Q., Li Y., Dong Y.L., Jiang B.J., Xi B.W., Qiang J. (2025). Integration of transcriptome, histopathology, and physiological indicators reveals regulatory mechanisms of largemouth bass (*Micropterus salmoides*) in response to carbonate alkalinity stress. Aquaculture.

[2] Yao Z.L., Lai Q.F., Zhou K., Rizalita R.E., Wang H. (2010). Developmental biology of medaka fish (*Oryzias latipes*) exposed to alkalinity stress. Appl. Ichthyol..

[3] Zhang Y., Wen H., Liu Y., Qi X., Sun D., Zhang C., Zhang K., Zhang M., Li J., Li Y. (2023). Gill histological and transcriptomic analysis provides insights into the response of spotted sea bass (*Lateolabrax maculatus*) to alkalinity stress. Aquaculture.

[4] Ondrasek G., Rengel Z. (2021). Environmental salinization processes: Detection, implications & solutions. Sci. Total Environ..

[5] Perry S.F., Gilmour K.M. (2006). Acid-base balance and CO_2_ excretion in fish: Unanswered questions and emerging models. Respirat Physiol. Neurobiol..

[6] Zhou H.S., Yao T.H., Zhang T.X., Sun M.Y., Ning Z.Y., Chen Y.Q., Mu W.J. (2024). Effects of chronic saline-alkaline stress on gill, liver and intestinal histology, biochemical, and immune indexes in Amur minnow (*Phoxinus lagowskii*). Aquaculture.

[7] Li X.J., Shen Y.D., Bao Y.G., Wu Z.X., Yang B.Q., Jiao L.F., Zhang C.D., Tocher D.R., Zhou Q.C., Zhou Q.C. (2022). Physiological responses and adaptive strategies to acute low-salinity environmental stress of the euryhaline marine fish black seabream (*Acanthopagrus schlegelii*). Aquaculture.

[8] Liu J., Zhang F.Y., Yan Z.H., Guo Z.Y., Lu Y.Q., Yao B.L., Li Y.H., Lv W.F. (2025). Effects of prolonged NaHCO_3_ exposure on the serum immune function, antioxidant capacity, intestinal tight junctions, microbiota, mitochondria, and autophagy in crucian carp (*Carassius auratus*). Ecotoxicol. Environ. Saf..

[9] Liu Y.J., Yao M.Z., Li S.W., Wei X.F., Ding L., Han S.C., Wang P., Lv B.C., Chen Z.X., Sun Y.C. (2022). Integrated application of multi-omics approach and biochemical assays provides insights into physiological responses to saline-alkaline stress in the gills of crucian carp (*Carassius auratus*). Sci. Total Environ..

[10] Liang P.P., Saqib H.S.A., Lin Z.Y., Zheng R.P., Qiu Y.T., Xie Y.T., Ma D.N., Shen Y.J. (2021). RNA-seq analyses of Marine Medaka (*Oryzias melastigma*) reveals salinity responsive transcriptomes in the gills and livers. Aquat. Toxicol..

[11] Linlin A., Zhang Y., Xu B., Zhang H., Li Y., Wang L., Liang J., Zhou W., Feng Z., Zhang H. (2024). Comprehensive analyses of annexins in naked carp (*Gymnocypris przewalskii*) unveil their roles in saline-alkaline stress. Aquaculture.

[12] Li Y., Gao P.C., Zhou K., Yao Z.L., Sun Z., Qin H.C., Lai Q.F. (2024). Effects of saline and alkaline stresses on the survival, growth, and physiological responses in juvenile mandarin fish (*Siniperca chuatsi*). Aquaculture.

[13] Lei C.X., Song H.R., Song H.M., Zhu T., Du J.X., Li S.J. (2024). RNA-seq and whole-genome re-sequencing reveal *Micropterus salmoides* growth-linked gene and selection signatures under carbohydrate-rich diet and varying temperature. Sci. Rep..

[14] Sun Y.C., Han S.C., Yao M.Z., Liu H.B., Wang Y.M. (2020). Exploring the metabolic biomarkers and pathway changes in crucian under carbonate alkalinity exposure using high-throughput metabolomics analysis based on UPLC-ESI-QTOF-MS. RSC Adv..

[15] Wu D., Wang L., Fan Z., Li J., Tang S., Zhao C., Zhang H., Zheng X. (2023). Comprehensive assessment of detoxification mechanisms of hydrolysis fish peptides in largemouth bass (*Micropterus salmoides*) under copper exposure: Tracing from bioaccumulation, oxidative stress, lipid deposition to metabolomics. Ecotoxicol. Environ. Saf..

[16] Lu Z., Huang W.Q., Wang S., Shan X.J., Ji C.L., Wu H.F. (2020). Liver transcriptome analysis reveals the molecular responses to low-salinity in large yellow croaker *Larimichthys crocea*. Aquaculture.

[17] Yu Q.R., Zhang F., Li R.N., Li E.C., Qin J.G., Chen L.Q., Wang X.D. (2025). Growth performance, antioxidant capacity, intestinal microbiota, and metabolomics analysis of Nile tilapia (*Oreochromis niloticus*) under carbonate alkalinity stress. Aquaculture.

[18] Sun Y., Wang S.L., Huang Y., Hong Y.H., Xu D.Y., Jiang C.W., Huang Z.Q. (2024). Unraveling the molecular mechanisms of nitrite-induced physiological disruptions in largemouth bass. Aquaculture.

[19] Zhang Y.C., Yu H.R., Chen H.P., Wang X.X., Tan Y.F., Sun J.L., Luo J., Song F.B. (2025). Integrative transcriptomic and metabolomic analyses reveal preliminary molecular mechanisms of gills response to salinity stress in *Micropterus salmoides*. Aquaculture.

[20] Shang X.C., Geng L.W., Wei H.J., Liu T.Q., Che X.H., Li W., Liu Y.H., Shi X.D., Li J.H., Teng X.H. (2024). Analysis revealed the molecular mechanism of oxidative stress-autophagy-induced liver injury caused by high alkalinity: Integrated whole hepatic transcriptome and metabolome. Front. Immunol..

[21] Wang Z.X., Pu D.C., Zheng J.S., Li P.Y., Lü H.J., Wei X.L., Li M., Li D.S., Gao L.H. (2023). Hypoxia-induced physiological responses in fish: From organism to tissue to molecular levels. Ecotoxicol. Environ. Saf..

[22] Li B.B., Wang G.X., Zheng X.Y., Zheng X.Y., Liu M.Y., Yang Y.C., Ren Y.Q., Zhang Y.T., Liu Y.F., He Z.W. (2025). Exposure to deltamethrin leads to gill liver damage, oxidative stress, inflammation, and metabolic disorders of Japanese flounder (*Paralichthys olivaceus*). Front. Toxicol..

[23] Chen S.Y., Wang F., Song J.L., Li F.X., Wang J.W., Jia Y.D. (2024). Sexual dimorphism in physiological responses of turbot (*Scophthalmus maximus* L.) under acute hypoxia and reoxygenation. Aquaculture.

[24] Wang X.X., Deng C.Y., Lin W.Y., Chen H.P., Yu H.R., Sun S.K., Sun J.L., Luo J., Song F.B. (2025). Transcriptome analysis revealed that largemouth bass (*Micropterus salmoides*) may mobilize liver lipid metabolism to provide energy for adaptation to hypertonic stress. Aquaculture.

[25] Askew K., Li K.Z., Olmos-Alonso A., Garcia-Moreno F., Liang Y.J., Richardson P., Tipton T., Chapman M.A., Riecken K., Beccari S. (2017). Coupled proliferation and apoptosis maintain the rapid turnover of microglia in the adult brain. Cell Rep..

[26] Tang J., Zhang Z.X., Miao J.J., Tian Y.M., Pan L.Q. (2022). Effects of benzo[*a*]pyrene exposure on oxidative stress and apoptosis of gill cells of Chlamys farreri in vitro. Environ. Toxicol. Pharmacol..

[27] Zhu G.H., Zhang L.P., Li Z.G., Wei A., Yang Y., Tian Y., Ma H.H., Wang D., Zhao X.X., Zhao Y.Z. (2020). Associations between *PRF1* Ala91Val polymorphism and risk of hemophagocytic lymphohistiocytosis: A meta-analysis based on 1366 subjects. World J. Pediatr..

[28] Buttini S., Cappellano G., Ripellino P., Briani C., Cocito D., Osio M., Cantello R., Dianzani U., Comi C. (2015). Variations of the perforin gene in patients with chronic inflammatory demyelinating polyradiculoneuropathy. Genes Immun..

[29] Das S., Tseng L.C., Chou C., Wang L., Souissi S., Hwang J.S. (2019). Effects of cadmium exposure on antioxidant enzymes and histological changes in the mud shrimp *Austinogebia edulis* (Crustacea: Decapoda). Environ. Sci. Pollut. Res..

[30] Estrada-Cárdenas P., Cruz-Moreno D.G., González-Ruiz R., Peregrino-Uriarte A.B., Leyva-Carrillo L., Camacho-Jiménez L., Quintero-Reyes I., Yepiz-Plascencia G. (2021). Combined hypoxia and high temperature affect differentially the response of antioxidant enzymes, glutathione and hydrogen peroxide in the white shrimp *Litopenaeus vannamei*. Comp. Biochem. Phys. A.

[31] Kim S., Jeon H., Bai S.C., Hur J.W., Han H. (2022). Effects of dietary supplementation with Arthrobacter bussei powder on growth performance, antioxidant capacity, and innate immunity of Pacific white shrimp (*Litopenaeus vannamei*). Aquac. Rep..

[32] Jing M., Wang Y.Q., Xu L.P. (2019). Andrographolide Derivative AL-1 Ameliorates Dextran Sodium Sulfate-Induced Murine Colitis by Inhibiting NF-κB and MAPK Signaling Pathways. Oxidative Med. Cell. Longev..

[33] Behl T., Rana T., Alotaibi G.H., Shamsuzzaman M., Naqvi M., Sehgal A., Singh S., Sharma N., Almoshari Y., Abdellatif A.A.H. (2022). Polyphenols inhibiting MAPK signalling pathway mediated oxidative stress and inflammation in depression. Biomed. Pharmacother..

[34] Gao H., Zhang J., Huang Z.Y., Zhang X., Rao Z.M., Xu M.J. (2025). The maintenance of redox homeostasis to regulate efficient glutathione metabolism in *Corynebacterium glutamicum*. Chem. Eng. J..

[35] Zhang R.Q., Shi X., Guo J.T., Mao X., Fan B.Y. (2024). Acute stress response in gill of Pacific white shrimp *Litopenaeus vannamei* to high alkalinity. Aquaculture.

[36] Bacila I.A., Elder C., Krone N. (2019). Update on adrenal steroid hormone biosynthesis and clinical implications. Arch. Dis. Child..

[37] Budin I., Szostak J.W. (2011). Physical effects underlying the transition from primitive to modern cell membranes. Proc. Natl. Acad. Sci. USA.

[38] Peterson B., Stovall K., Monian P., Franklin J.L., Cummings B.S. (2008). Alterations in phos-pholipid and fatty acid lipid profiles in primary neocortical cells during oxidant-induced cell injury. Chem. Biol. Interact..

[39] Wu X.Y., Zou X.J., Chang Q., Zhang Y.R., Li Y.H., Zhang L.Q., Huang J.F., Liang B. (2013). The Evolutionary Pattern and the Regulation of Stearoyl-CoA Desaturase Genes. Biomed. Res. Int..

[40] Crovetto C.A., Córdoba O.L. (2016). Structural and biochemical characterization and evolutionary relationships of the fatty acid-binding protein 10 (Fabp10) of hake (*Merluccius hubbsi*). Fish. Physiol. Biochem..

[41] Vouilloz A., Bourgeois T., Diedisheim M., Pilot T., Jalil A., Le Guern N., Bergas V., Rohmer N., Castelli F., Leleu D. (2025). Impaired unsaturated fatty acid elongation alters mitochondrial function and accelerates metabolic dysfunction-associated steatohepatitis progression. Metab. Clin. Exp..

[42] Liu X.L., Li H.Y., Dong H., Wang P.P., Li Y.T., Wu K.G. (2023). Effect of cinnamon essential oil dietary supplementation on the growth, fatty acid composition, and meat quality of tilapia. J. Food Sci..

[43] Zheng T., Song Z., Qiang J., Tao Y.F., Zhu H.J., Ma J.L., Xu P. (2021). Transport Stress Induces Skin Innate Immunity Response in Hybrid Yellow Catfish (*Tachysurus fulvidraco*♀ × *P. vachellii*♂) Through TLR/NLR Signaling Pathways and Regulation of Mucus Secretion. Front. Immunol..

[44] Qiang J., Tao Y.F., Zhu J.H., Lu S.Q., Cao Z.M., Ma J.L., He J., Xu P. (2022). Effects of heat stress on follicular development and atresia in Nile tilapia (*Oreochromis niloticus*) during one reproductive cycle and its potential regulation byautophagy and apoptosis. Aquaculture.

[45] Parkhomchuk D., Borodina T., Amstislavskiy V., Banaru M., Hallen L., Krobitsch S., Lehrach H., Soldatov A. (2009). Transcriptome analysis by strand-specific sequencing of complementary DNA. Nucleic Acids Res..

[46] Shao C.W., Bao B.L., Xie Z.Y., Chen X.Y., Li B., Jia X.D., Yao Q.L., Ortí G., Li W.H., Li X.H. (2017). The genome and transcriptome of Japanese flounder provide insights into flatfish asymmetry. Nat. Genet..

[47] Pertea M., Pertea G.M., Antonescu C.M., Chang T.C., Mendell J.T., Salzberg S.L. (2015). StringTie enables improved reconstruction of a transcriptome from RNA-seq reads. Nat. Biotechnol..

[48] Wang A., Li S.L., Liu Y.J., Han Z.H., Chen N.S. (2020). The inclusion of fenugreek seed extract aggravated hepatic glycogen accumulation through reducing the expression of genes involved in insulin pathway and glycolysis in largemouth bass, *Micropterus salmoides*. Aquaculture.

[49] Anders S., Huber W. (2010). Differential expression analysis for sequence count data. Genome Biol..

[50] Yu G.C., Wang L.G., Han Y.Y., He Q.Y. (2012). clusterProfiler: An R Package for Comparing Biological Themes Among Gene Clusters. Omics.

[51] Livak K.J., Schmittgen T.D. (2001). Analysis of relative gene expression data using real-time quantitative PCR and the 2^–∆∆CT^ method. Methods.

[52] Gu Z.G. (2022). Complex heatmap visualization. iMeta.

[53] Dunning M.J., Smith M.L., Ritchie M.E., Tavaré S. (2007). Beadarray: R classes and methods for Illumina bead-based data. Bioinformatics.

