# Supplementary material for: Integrated Transcriptomic and Metabolomic Analyses Reveal Physiological and Hepatic Metabolic Responses of Largemouth Bass (Micropterus salmoides) to Subacute Saline–Alkaline Stress
Source: Int J Mol Sci. 2025 Dec 16;26(24):12091. doi: 10.3390/ijms262412091 (PMC12733285; doi:10.3390/ijms262412091)
Supplement: Supplementary file 1 [file ijms-26-12091-s001.zip › ijms-4012201-supplementary.pdf]

Table S1. Overview of transcriptome sequencing reads and quality filtering of largemouth bass.

Sample	Raw Reads	Clean Reads	Clean Bases (G)	Error Rate (%)	Q20 (%)	Q30 (%)	GC pct (%)
NC-1	52043048	50394294	7.56	0.01	97.98	93.67	47.74
NC-2	46445854	45626076	6.84	0.01	97.90	93.51	48.49
NC-3	56797354	55949880	8.39	0.01	98.03	93.88	49.09
NC-4	57428378	56388842	8.46	0.01	98.09	94.04	48.92
NC-5	70840998	69318882	10.4	0.01	97.94	93.58	48.93
NC-6	51778936	50894164	7.63	0.01	99.01	96.74	49.22
SA-1	59541624	58402546	8.76	0.01	98.10	94.08	48.36
SA-2	49531688	48538926	7.28	0.01	97.91	93.53	48.06
SA-3	56061876	55085176	8.26	0.01	98.22	94.43	48.16
SA-4	59920660	58984402	8.85	0.01	98.13	94.15	48.99
SA-5	49643924	48728274	7.31	0.01	97.94	93.61	48.16
SA-6	67475398	66252668	9.94	0.01	98.13	94.18	48.78
